# Impacts of Maternal Preeclampsia Exposure on Offspring Neuronal Development: Recent Insights and Interventional Approaches

**DOI:** 10.3390/ijms252011062

**Published:** 2024-10-15

**Authors:** He Zhang, Jinju Lin, Huashan Zhao

**Affiliations:** 1 Department of Pathophysiology, College of Basic Medical Sciences, Dalian Medical University, Dalian 116044, China; 2 Center for Energy Metabolism and Reproduction, Shenzhen Institutes of Advanced Technology, Chinese Academy of Sciences, Shenzhen 518055, China; jinju_lin@126.com

**Keywords:** preeclampsia, offspring, neurological outcomes, neuronal abnormalities, intervention

## Abstract

Preeclampsia, a hypertensive disorder during pregnancy, frequently correlates with adverse neurological outcomes in offspring, including cognitive impairments, autism spectrum disorder, depressive disorder, attention deficit hyperactivity disorder, and cerebral palsy. Despite these known consequences, the understanding of neuronal damage in the offspring of preeclamptic mothers remains insufficient. Here, we review the neuronal abnormalities resulting from maternal preeclampsia exposure, which include disrupted neurogenesis, loss of neuronal cell integrity, accumulation of cellular debris, decreased synaptogenesis and myelination, and increased neurite growth stimulated by maternal preeclampsia serum. The underlying mechanisms potentially driving these effects involve microglial activation, inflammatory responses, and reduced angiogenesis. Intervention strategies aimed at improving fetal neuronal outcomes are also discussed, encompassing pharmacological treatments such as pravastatin, tadalafil, and melatonin, as well as non-pharmacological approaches like dietary modifications, maternal exercise, and standard care for children. These interventions hold promise for clinical application, offering avenues to address early neuronal abnormalities and prevent the onset of long-term neurological disorders.

## 1. Introduction

Preeclampsia is a common pregnancy complication that manifests as high blood pressure occurring after 20 weeks of pregnancy, accompanied by maternal organ damage, like pulmonary edema and kidney or liver dysfunction [1,2]. The pathophysiological mechanisms of preeclampsia involve placental dysfunction [3] and are characterized by oxidative stress [4,5], ferroptosis [6,7], and inflammation [8,9]. Worldwide, approximately 4 million women are diagnosed with preeclampsia each year, resulting in the deaths of 70,000 women and 500,000 infants [10]. Survivors of preeclampsia have a shortened life expectancy and an increased risk of stroke, cardiovascular disease, and diabetes [11], while infants from preeclamptic pregnancies face neurodevelopmental delays and susceptibility to cardiovascular and metabolic diseases [12,13].

The developmental origins of health and disease (DoHaD) concept posits that environmental factors, particularly those encountered during critical periods of early development (prenatal and early postnatal), can have long-lasting effects on an individual’s risk of developing chronic diseases in adulthood [14]. According to the DoHaD concept, the hypoxic and ischemic uterine environment resulting from preeclampsia may lead to enduring alterations in the development and function of the fetal nervous system [15]. Consequently, offspring born from preeclamptic pregnancies face heightened susceptibility to neurological disorders, including cognitive impairments, autism spectrum disorder (ASD), depressive disorder, attention deficit hyperactivity disorder (ADHD), and cerebral palsy, often rooted in aberrations of fetal neuronal development [16]. Although advances have been made in the study of preeclampsia in recent decades, little is known about its impacts on offspring neuronal abnormalities. Understanding how preeclampsia affects the neuronal development of the fetus is critical to improving the offspring’s neuronal development during pregnancy. The purpose of this review is to explore the long-term neurodevelopmental consequences of prenatal exposure to preeclampsia, assess its impacts on offspring neuronal development and potential mechanisms, explore interventional approaches, and possibly provide insights for future research directions and clinical practice.

## 2. Long-Term Neurodevelopmental Consequences of Prenatal Preeclampsia Exposure

**Figure 1:**
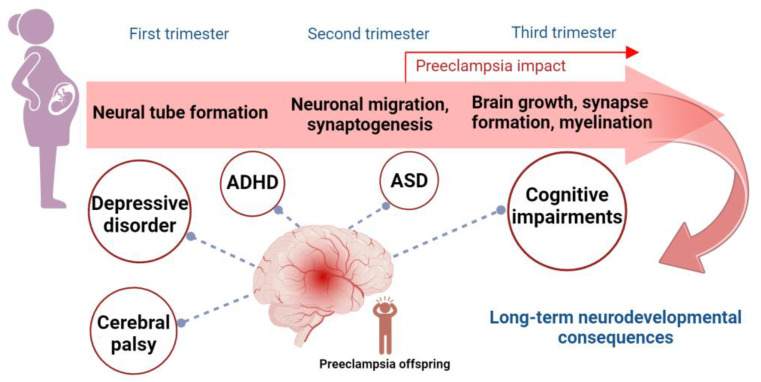
Key processes in brain development and long-term neurodevelopmental outcomes of prenatal preeclampsia exposure. Critical processes in brain development during the three trimesters of pregnancy can be impacted by prenatal exposure to preeclampsia. These processes include neural tube formation in the first trimester, neuronal migration and synaptogenesis in the second trimester, and brain growth, synapse formation, and myelination in the third trimester. Preeclampsia, which typically occurs after 20 weeks of pregnancy (in the second trimester), is associated with an increased risk of long-term neurodevelopmental consequences in offspring. These include cognitive impairments, ASD, ADHD, depressive disorder, and cerebral palsy. Abbreviations: ASD, autism spectrum disorder; ADHD, attention deficit hyperactivity disorder.

The fetal to neonatal period represents a pivotal phase in brain development, marked by rapid neurodevelopmental processes pivotal for establishing fundamental neural circuits in the brain [17]. Prenatal exposure to preeclampsia poses a significant risk of disrupting the development of the fetal nervous system, potentially leading to poor children’s neurodevelopment outcomes (Figure 1).

### 2.1. Cognitive Impairments

Preeclampsia has been consistently linked to cognitive impairments in offspring, with evidence suggesting both immediate and long-term effects on neurodevelopment. Studies indicate that children born to mothers with preeclampsia face a heightened risk of intellectual disability, cognitive impairment, and lower intelligence quotient (IQ) scores [18,19,20]. Specifically, children born small for gestational age (SGA) to preeclamptic mothers exhibit lower IQ scores compared to those born to non-preeclamptic mothers, even when accounting for gestational age [21]. These cognitive deficits appear early, as evidenced by children from severe preeclamptic pregnancies failing in at least one developmental category on the Ages and Stages Questionnaire up to age 3, highlighting the risk for neurodevelopmental delays [22].

Furthermore, preeclampsia has been associated with specific cognitive domains, such as working memory and oculomotor control, with offspring showing impairments in these areas compared to those from uncomplicated pregnancies, as revealed through psychometric and eye-tracking assessments [23]. The compounded effect of preeclampsia with intrauterine growth restriction (IUGR) intensifies the cognitive deficits, with children exhibiting lower verbal and full-scale IQs compared to children not exposed to preeclampsia or those with appropriate birth weights [24].

The long-term impacts of prenatal exposure to preeclampsia extend into adolescence and adulthood. Children exposed to preeclampsia consistently score lower in mathematics across different grade levels at ages 9, 12, and 15 [25], while males display reduced cognitive performance in adulthood [26], suggesting that the effects of preeclampsia on cognitive abilities may be enduring. However, this relationship is not uniform, as one study reports that maternal preeclampsia does not impair cognitive performance in offspring who reach late adolescence (17 years old), though it increases the risk of hypertension [27].

### 2.2. Autism Spectrum Disorder (ASD)

Perinatal conditions, particularly birth asphyxia and preeclampsia, have been associated with an increased risk of childhood ASD, even after adjusting for gestational age and other potential confounders [28]. A population-based study of singleton live births in Sweden from 1982 to 2010 reveals a connection between preeclampsia and increased ASD risk, especially among SGA infants [29]. Similarly, research from Norway identifies preeclampsia and related conditions (such as eclampsia) as risk factors for ASD, ADHD, and epilepsy [30]. Further evidence emphasizes the role of preeclampsia in ASD risk by linking it to a low birth weight [31]. The Childhood Autism Risks from Genetics and the Environment (CHARGE) study finds that children with ASD are twice as likely to have been exposed to maternal preeclampsia in utero after adjusting for maternal education and pre-pregnancy obesity [32]. These findings, supported by three meta-analyses, underscore maternal preeclampsia as a perinatal risk factor that substantially increases the likelihood of ASD in offspring [33,34,35].

However, contrasting evidence exists. One study presents an unexpected finding, suggesting that preeclampsia may have a protective effect in children with ASD, particularly among those without intellectual disabilities [36]. This highlights the complex nature of the relationship between preeclampsia and ASD, where outcomes may vary depending on specific factors.

### 2.3. Depressive Disorder

A retrospective analysis from the Helsinki Birth Cohort reveals that individuals born after a primiparous pregnancy complicated by preeclampsia exhibit over 30% higher rates of depressive symptoms in adulthood compared to those born after normotensive pregnancies, even after adjusting for confounding factors such as maternal age and BMI at delivery [37]. Further research by the same cohort demonstrates that the psychiatric and psychological effects of preeclampsia persist for up to seven decades, suggesting a lasting impact across the lifespan [38]. The connection between preeclampsia and mental health outcomes is also supported by a study tracking 538 adults, which finds that FGR and preeclampsia are associated with an increased risk of major depressive disorder and cardiovascular disease later in life, with women being particularly affected [39]. Interestingly, while pregnancy-related hypertension without proteinuria is associated with a higher risk of severe mental disorders in adult offspring, preeclampsia itself appears to confer a lower risk of such disorders in male offspring [40], which suggests the intricate and potentially sex-specific long-term effects of preeclampsia on both the mental and physical health of offspring.

### 2.4. Attention Deficit Hyperactivity Disorder (ADHD)

Maternal preeclampsia as a risk factor for ADHD in offspring has been supported across different populations and methodologies. For example, a population-based case-control study in Western Australia [41] and research from the Millennium Cohort Study (MCS) [42] both demonstrate that preeclampsia is associated with an elevated risk of ADHD in offspring. Maternal genitourinary infections, in conjunction with preeclampsia, heighten the odds of ADHD, suggesting that comorbid maternal conditions exacerbate this risk [43]. A similar result is reported in a large cohort study using data from over 7200 children in the Avon Longitudinal Study of Parents and Children, which finds that maternal preeclampsia nearly triples the risk of ADHD in offspring at ages 7 and 10, as confirmed through log-binomial regression and generalized estimating equation models [44]. A meta-analysis synthesizes these findings, confirming that intrauterine exposure to preeclampsia consistently increases the risk of ADHD across studies [34]. Collectively, maternal preeclampsia exposure is likely to increase the risk of ADHD in offspring.

### 2.5. Cerebral Palsy

Cerebral palsy, a prevalent childhood condition marked by permanent movement and postural impairments due to early brain damage, is intricately linked to various prenatal factors [45]. Among these, preeclampsia, a major cause of preterm birth, nearly doubles the risk of cerebral palsy in children, particularly when it is diagnosed before 37 weeks of gestation, with the risk remaining elevated even for full-term infants [46,47]. This increased risk is largely mediated through complications such as preterm birth and FGR, as evidenced by a cohort study linking data from the Norwegian Cerebral Palsy Registry and the Medical Birth Registry, which identifies a heightened risk of cerebral palsy in term-born children who are also SGA [48]. In addition, a population-based cohort study from Denmark links preeclampsia to a higher risk of epilepsy, specifically in children born after 37 weeks of gestation [49]. There is a complex interplay between preeclampsia, gestational age, and fetal growth in determining risks for cerebral palsy. While preeclampsia is traditionally associated with preterm birth, its contribution to the risk of cerebral palsy appears to extend beyond preterm birth alone, likely involving additional mechanisms.

## 3. Neuronal Abnormalities Arising from Maternal Preeclampsia Exposure

**Figure 2:**
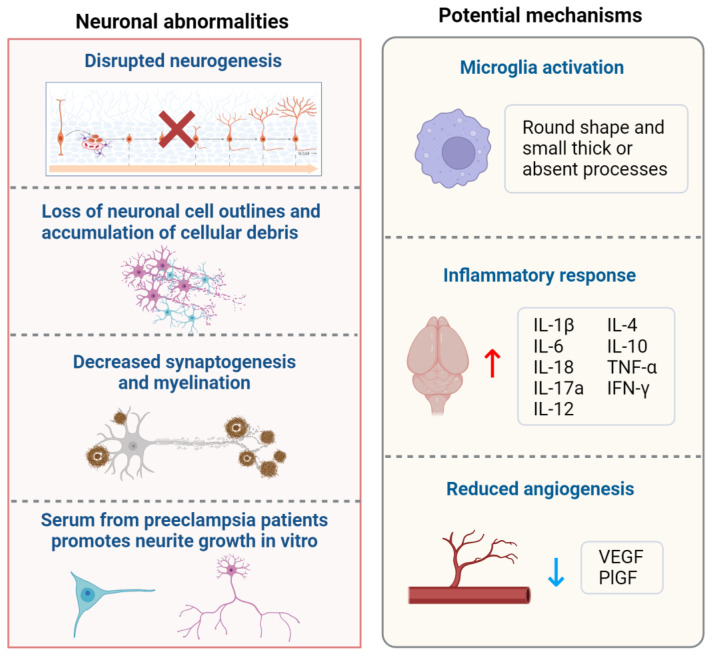
Neuronal abnormalities and potential mechanisms of maternal preeclampsia exposure. Offspring neuronal development is impacted by maternal preeclampsia exposure, leading to disrupted neurogenesis, loss of neuronal cell integrity, accumulation of cellular debris, decreased synaptogenesis and myelination, and increased neurite growth stimulated by maternal preeclampsia serum. Potential mechanisms underlying these effects include microglial activation (evidenced by a round shape and small, thick, or absent processes), inflammatory responses (characterized by increased levels of IL-1β, IL-6, IL-18, IL-17a, IL-12, IL-4, IL-10, TNF-α, and IFN-γ), and reduced angiogenesis (reflected by decreased levels of VEGF and PlGF). Abbreviations: IL, interleukin; TNF-α, tumor necrosis factor-α; IFN-γ, interferon-gamma; VEGF, vascular endothelial growth factor; PlGF, placental growth factor; ↑, increase; ↓, decrease.

Neurodevelopment is primarily established during the embryonic period, orchestrating crucial processes in neuronal and brain structure formation [50]. However, ethical and legal constraints render direct research involving human fetuses impractical, necessitating the utilization of animal models to unravel neuronal abnormalities and underlying mechanisms. The sequence of neurogenesis exhibits conservation across diverse mammalian species [51], encompassing critical processes like neural tube formation, neuronal migration, synaptogenesis, rapid brain growth, synapse formation, and myelination [52]. While mammals like rats, mice, and sheep are often used as animal models to study the effects of preeclampsia on offspring neuronal development, it is important to acknowledge the limitations inherent in these models. Each species has distinct differences in physiological and placental development that can influence the applicability of findings to human preeclampsia [53]. Consequently, although these models provide valuable insights, caution must be exercised when interpreting the data in the context of human conditions [54]. The neuronal alterations observed in offspring exposed to preeclampsia are reviewed below (Figure 2, left part).

### 3.1. Disrupted Neurogenesis

Soluble fms-like tyrosine kinase 1 (sFlt-1), a soluble form of the vascular endothelial growth factor receptor, emerges as a pivotal player associated with the onset of preeclampsia [55,56]. In a mouse model of preeclampsia induced by an adenovirus carrying sFlt-1 via tail vein injection, male and female offspring exhibit distinct, sex-specific alterations in brain region volumes via whole-brain magnetic resonance imaging, indicating changes in neuroanatomic programming [57].

N(ω)-nitro-L-arginine methyl ester (L-NAME), a potent nitric oxide (NO) synthase inhibitor, is widely used to induce preeclampsia in animal models due to its role in disrupting NO production, which is essential for vasodilation and thrombosis inhibition [58,59]. In a rat preeclampsia model induced by L-NAME injection, an inadequate proliferation of neural progenitor cells leads to a reduced brain weight and volume at birth, as evidenced by a decrease in BrdU-positive cells in the neocortex’s ventricular zone and a drop in *Fgf2* expression, a gene essential for neurogenesis [60]. Intriguingly, water maze tests unveiled the impaired spatial learning and memory abilities in the offspring of these rats, corroborating the findings from cohort studies [61]. These deficits suggest that compromised hippocampal neurogenesis may contribute to the observed cognitive impairments.

### 3.2. Loss of Neuronal Cell Outlines and Accumulation of Cellular Debris

Deoxycorticosterone acetate (DOCA), a synthetic corticosteroid hormone mirroring adrenal cortex hormones, serves as a crucial tool in inducing hormonal imbalance in vivo [62]. DOCA administration to animals effectively simulates hormonal dysregulation, inhibiting placental hormone production, inducing vasoconstriction, and precipitating renal dysfunction, thereby mimicking the clinical manifestations of preeclampsia [63]. Induction of the preeclampsia rat model via an intraperitoneal injection of DOCA reveals necrotic areas in the cerebral cortex and hippocampus, which exhibit loss of neuronal cell outlines, accumulation of cellular debris, atrophic glial cells, and vacuolated neurons, along with a reduction in neuronal counts [64], suggesting preeclampsia may exert neurotoxic effects, shedding light on its detrimental impact on fetal brain health and proposing a mechanistic link through which the condition may contribute to neurological disorders.

### 3.3. Decreased Synaptogenesis and Myelination

In an L-NAME-induced preeclampsia mouse model, fluorescent immunohistochemistry of the offspring brain reveals a decrease in synaptophysin expression in the hippocampus at postnatal days 15 (P15) and P30 and a reduced myelin basic protein expression in the cingulum, suggesting impaired synaptogenesis and myelination [65]. The reduction in oligodendrocyte transcription factor 2 expression in the offspring’s brain from pregnancy day 19 until P60, along with increased latency to locate the platform in the Morris water maze, prolonged time to traverse the balance beam, and decreased hanging time on the wire test, are observed in the preeclampsia rat model [66]. These two rodent studies imply that preeclampsia may lead to long-term sensorimotor and cognitive deficits through decreased synaptogenesis and demyelination.

### 3.4. Serum from Preeclampsia Patients Promotes Neurite Growth In Vitro

In primary cortical neurons, the addition of the 3% serum isolated from preeclampsia patients induces increased neuronal growth and branching [67], highlighting a potential link between the serum’s composition and neurodevelopmental processes. Similarly, the 3% preeclampsia serum promotes neurite growth in the human neural progenitor cell line (SH-SY5Y) [68], suggesting that the serum contains factors that enhance neuronal growth and neurite development. This implies that preeclampsia may involve mechanisms extending beyond vascular and placental dysfunction, impacting neural development in the fetus. The presence of bioactive factors in the serum that promote neural growth and development raises questions about their identities and specific roles in the context of preeclampsia. These factors could be proteins, cytokines, or other molecules that interact with neural cells, possibly contributing to the adaptive or maladaptive responses observed in preeclampsia. For example, maternal inflammatory factors, represented by interleukin-6 (IL-6), may lead to cognitive and behavioral deficits in offspring by altering synaptic formation and affecting synaptic function [69]. Although in vitro stimulation provides valuable cues, the current understanding of how bioactive factors from maternal circulation promote neurite growth remains limited. Further investigation is essential to identify and characterize these biological factors, understand their molecular pathways, and elucidate their precise roles in the pathophysiology of preeclampsia, which could uncover novel insights into how preeclampsia affects the nervous system and provide new therapeutic strategies.

## 4. Potential Mechanisms Underlying the Risk of Neurodevelopment Alterations in Offspring of Preeclampsia

Several pertinent reviews have addressed the mechanisms by which preeclampsia affects neuronal development in offspring, focusing on maternal factors such as placental ischemia, oxidative stress, immune dysregulation, and inflammation [2,15,16,70,71,72]. However, no review to date has concentrated on the mechanisms specifically related to fetal changes. Thus, we discuss them in the following sections (Figure 2, right part).

### 4.1. Microglia Activation

The offspring of a preeclampsia mouse model exhibit increased microglia activation in the brain, characterized by a round shape and small, thick, or absent processes, which are observed through immune affinity purification from the P10 brains [73]. Microglia play a vital role in regulating neurogenesis and brain development [74,75]. The increased activation of microglia suggests an ongoing immune response and potential neuronal damage in preeclampsia offspring, which may contribute to neurodevelopmental deficits.

### 4.2. Inflammatory Response

Increased fetal demise, cerebral microbleeds, and elevated levels of pro-inflammatory cytokines, such as IL-1β, IL-6, and IL-18, alongside a decrease in microglial density in the subventricular zone, are observed in a preeclampsia rat model, which suggests that preeclampsia leads to microvascular dysfunction, neuroinflammation, and reduced microglial presence in critical brain areas [76]. Analysis of fetal brains on embryonic day 17.5 from a preeclampsia mouse model induced by angiotensin II (Ang II) reveals an elevated expression of inflammatory cytokines, including IL-6, IL-17a, tumor necrosis factor-α (TNF-α), interferon-gamma (IFN-γ), IL-12, IL-4, and IL-10, with microglia in the fetal brains transforming into an activated, amoeboid morphology, indicating heightened inflammatory responses [77]. Together, maternal preeclampsia may alter inflammatory conditions in the fetal brain, potentially leading to subsequent brain dysfunction in the offspring.

### 4.3. Reduced Angiogenesis

A reduced blood vessel area is observed in the motor and somatosensory cortex of offspring at P5 from a preeclampsia-like syndrome (PELS) mouse model, coinciding with lower circulating levels of vascular endothelial growth factor (VEGF) and placental growth factor (PlGF), which lead to impaired angiogenic capacity, reduced cell migration, abnormal F-actin filament assembly, and fewer filopodia in brain endothelial cells [78]. In a reduced uterine perfusion pressure (RUPP) induced preeclampsia mouse model, reductions in brain microvascular perfusion and reactivity to external stimuli are noted in the offspring at P5, while RUPP surgery results in a 40–50% decrease in uterine blood flow, leading to unfavorable perinatal outcomes; the angiogenic proteomic profile in the brains of offspring indicates changes related to inflammation, apoptosis, cancer, and cellular senescence [79]. Thus, impaired brain perfusion and altered angiogenic protein expression likely contribute to neurovascular and cognitive challenges in the offspring of preeclamptic pregnancies.

## 5. Interventional Approaches

**Figure 3:**
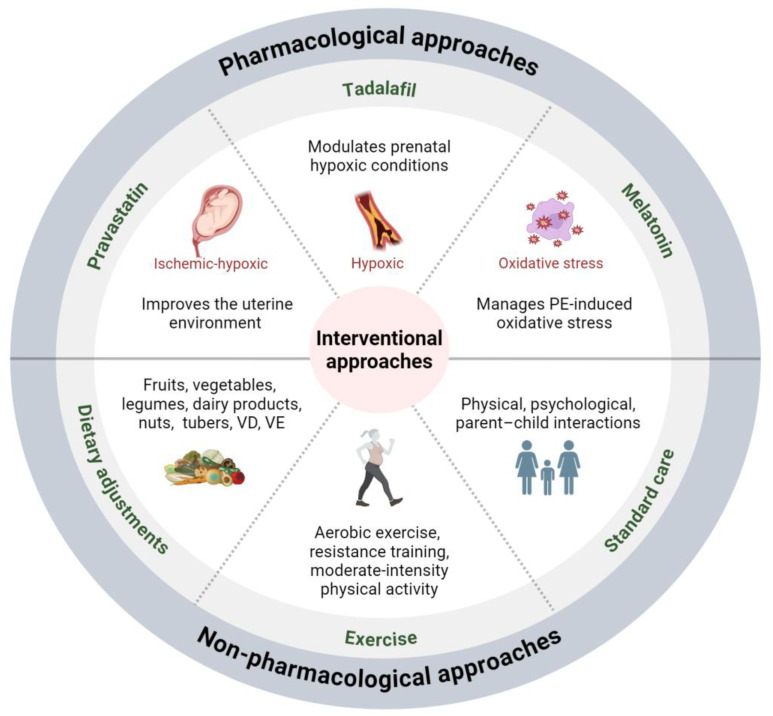
Interventional approaches for mitigating the impact of maternal preeclampsia on offspring neuronal development. Pharmacological interventions, including pravastatin, tadalafil, and melatonin, alongside non-pharmacological approaches, such as dietary adjustments and exercise for mothers and standard care for children, offer potential avenues for preventing and mitigating the impact of maternal preeclampsia on offspring neuronal development. Abbreviations: PE, preeclampsia; VD, vitamin D; VE, vitamin E.

Current reviews predominantly emphasize treatments that alleviate symptoms and complications for mothers [72,80,81]. In this review, we present the intervention strategies designed to mitigate the effects of prenatal preeclampsia on neuronal development in offspring (Figure 3). Regarding the pharmacological approaches, three drugs have been proven effective in correcting neuronal abnormalities in offspring from murine models, with clinical trials for preeclampsia reported (Table 1).

### 5.1. Pharmacological Approaches

#### 5.1.1. Pravastatin

Pravastatin, an HMG-CoA (3-Hydroxy-3-Methyl-Glutaryl-Coenzyme A) reductase inhibitor commonly prescribed for reducing cholesterol levels in the bloodstream [82], has drawn attention to its potential to alleviate the detrimental impacts of preeclampsia on both maternal and fetal health [83,84,85]. Recent research underscores its efficacy in improving endothelial function and reducing inflammation and oxidative stress [86]. Antenatal treatment with pravastatin in animal models of preeclampsia leads to improved blood pressure, enhanced vascular reactivity, and increased pup growth [87,88]. Moreover, pravastatin treatment in a preeclampsia mouse model can normalize apoptotic pathways, neuronal migration, and myelin inflammatory pathways in the offspring’s frontal cortex and hypothalamus [89]. Preeclampsia alters sex-specific brain development, causing deficits in balance and coordination in adult offspring, with maternal pravastatin treatment showing potential for prevention [57,90].

The phase I clinical trial of pravastatin (10 mg) establishes preliminary safety and pharmacokinetic data for its use in preventing preeclampsia in high-risk pregnant women [91]. Building on this, a follow-up randomized pilot trial administering a daily dose of 20 mg from 12 + 0 to 16 + 6 weeks of gestation until delivery demonstrates more favorable pregnancy and neonatal outcomes, with headaches, heartburn, and musculoskeletal pain as the most common side effects [92]. An open-label multicenter randomized clinical trial confirms that pravastatin (20 mg) reduces the risk of preterm preeclampsia and preterm birth in high-risk women [93]. While three clinical trials could not definitively establish the benefits of pravastatin for preventing preeclampsia [94], they provide valuable insights into its potential efficacy.

Due to its hydrophilic nature and limited placental permeability, pravastatin’s capacity to enhance the development of offspring affected by preeclampsia is attributed not to the direct effects on the offspring but rather to its ability to ameliorate the intrauterine environment [90]. To sum up, pravastatin could be a promising therapeutic strategy for mitigating the adverse effects of preeclampsia, ultimately improving both maternal and fetal outcomes.

#### 5.1.2. Tadalafil

NO activates guanylate cyclase to increase the cyclic guanosine monophosphate (cGMP) levels, thereby regulating placental vascular tone [95,96]. Molecular mediators of the NO-dependent pathways, including cGMP-specific phosphodiesterases (PDEs), are expressed in placental blood vessels [97]. PDEs degrade cGMP, converting it into inactive 5′-GMP, with specific subtypes like PDE5, prevalent in vascular smooth muscle cells, playing a crucial role in cGMP degradation [98]. Tadalafil, a selective PDE5 inhibitor, demonstrates therapeutic potential in preeclampsia based on findings from a small clinical trial [99]. An animal study shows that it reduces hypoxia-inducible factor-2α expression in both the placenta and fetal brain while also promoting synaptogenesis and myelination in offspring at P15 and P30 [65]. These preclinical investigations indicate that tadalafil may mitigate the adverse effects of preeclampsia on fetal growth and neuronal development.

The phase I clinical trial of tadalafil for women with preeclampsia demonstrates no maternal adverse events associated with the 10 mg/day dose. In comparison, grade 1 headaches occurred in two cases, and one case of grade 1 palpitation also occurred using the 20 mg/day dose, and only one instance of grade 1 headache is noted at the 40 mg/day dose, all of which spontaneously resolve within three days without any fetal adverse events [100]. The phase II clinical trial indicates that tadalafil (20 mg/day) does not increase the incidence of maternal or neonatal adverse events, including intrauterine and neonatal deaths, nor does it prolong the gestational period in pregnant women with hypertensive disorders of pregnancy [101]. Although these are only two clinical trials, they suggest that tadalafil may be safe for women with preeclampsia and their offspring.

#### 5.1.3. Melatonin

Melatonin (N-acetyl-5-methoxytryptamine), a hormone secreted by the pineal gland and the placenta during pregnancy, has the unique ability to cross both the placenta and the blood–brain barrier [102]. Commonly referred to as the “sleep hormone”, melatonin also exhibits antioxidant properties, effectively neutralizing free radicals and mitigating cellular oxidative stress [103,104,105]. This function makes melatonin a candidate for therapeutic intervention in preeclampsia [106,107]. Melatonin treatment in a preeclampsia rat model enhances fetal and fetal brain weight, reduces fetal mortality, increases brain superoxide dismutase levels, reduces brain malondialdehyde (MDA) levels, and increases brain TGF-β, with the melatonin-treated group showing normal glial cells and neuropil, whereas the untreated group exhibits a loss of neuronal cell outlines and accumulation of cellular debris [64].

The safety of melatonin treatment for both mothers and fetuses has been established [108,109]. Melatonin administration prolongs the diagnosis-to-delivery interval by an average of 6 days and reduces the need for increased antihypertensive medication compared to the controls, and it does not affect other clinical or biochemical measures of disease severity [110]. However, there are currently no clinical trials assessing antenatal maternal melatonin supplementation for fetal neuroprotection in preeclampsia. Thus, melatonin’s neuroprotective effects have only been investigated in preclinical studies, highlighting its potential as a therapeutic intervention to mitigate neurological damage in offspring.

**Table 1:** Summary of pharmacological interventions for altered neuronal development in offspring from preeclampsia animal models and clinical trials for preeclampsia treatment.

Intervention	Animal Model and Administration	Function	Mechanism	Clinical Trial for Preeclampsia
Pravastatin	CD-1 mice, tail vein injection with adenovirus carrying sFlt-1; 5 mg/kg/day through drinking water	Restores the normal development of pathways for apoptosis, neuronal migration, and myelin inflammation in the frontal cortex and hypothalamus [89]	N/A	20 mg twice daily starting from 14 to 20 weeks gestation until delivery [93]; 10 mg/day orally between 12^0/7^ and 16^6/7^ weeks of gestation; no identifiable safety risks [91]; * 20 mg/day orally from 12 + 0 to 16 + 6 weeks gestation; adverse events: headache, heartburn, musculoskeletal pain [92]
	CD-1 mice, tail vein injection with adenovirus carrying sFlt-1; 5 mg/kg/day through drinking water	Improves brain volumes and cortical cell counts in 6 months offspring brain [57]	N/A	
	CD-1 mice, tail vein injection with adenovirus carrying sFlt-1; 5 mg/kg/day through drinking water	Improves adult offspring vestibular function, balance, and coordination [90]	Improves the uterine environment	
Tadalafil	C57BL/6 mice, L-NAME dissolved in 0.5% CMC in drinking water; 0.08 mg/mL in 0.5% CMC in drinking water	Improves synaptogenesis and myelination in offspring [65]	Modulates prenatal hypoxic conditions	No maternal adverse events at 10 mg/day; grade 1 headache and palpitation at 20 mg/day; grade 1 headache at 40 mg/day; all adverse events resolve spontaneously within 3 days [100]; 20 mg orally between 20 and 33 weeks of gestation; adverse events do not occur [101]
Melatonin	*Rattus norvegicus*, injection DOCA; 10 mg/kg body weight; gavage	Improves fetal brain weight; reduces fetal death rate and brain MDA; increases brain superoxide dismutase and TGF-β [64]	Manages oxidative stress in the placenta and fetal cerebral cortex	3 mg orally before cesarean section [108]; 10 mg three times a day; safe for mothers and their fetuses [110]

* In this study, all newborns passed their brainstem auditory-evoked response potential or similar hearing screening tests.

### 5.2. Non-Pharmacological Management Approaches

Dietary adjustments, exercise, and standard care of children are three essential non-pharmacological approaches to improving maternal and child health. Dietary recommendations emphasize the consumption of fruits, vegetables, legumes, dairy products, nuts, and tubers [111,112,113], along with vitamin D [114,115,116] and vitamin E [117,118,119] supplementation, all of which have shown efficacy in preeclampsia prevention. Regular exercise initiated before 24 weeks of gestation can reduce the risk of preeclampsia by up to 40% [120], suggesting a potential low-cost intervention to improve prenatal care for at-risk women [121]. Exercise may ameliorate preeclampsia by promoting angiogenesis and improving placental function; specifically, regular physical activity during pregnancy is associated with elevated serum PlGF levels and reduced concentrations of sFlt-1 and soluble endoglin (sEng) in late gestation [122]. Additionally, exercise training enhances the maternal and possibly fetal plasma volume, increases the intervillous space blood volume, and boosts cardiac output, all of which contribute to better placental health [123]. However, determining the optimal type and intensity of exercise remains challenging due to the diverse approaches studied.

In terms of offspring, the first three years are pivotal for neurodevelopment, with events during this period significantly impacting cognitive and social abilities [124]. Exposure to preeclampsia during gestation can adversely affect a child’s neurodevelopment, necessitating measures to mitigate its consequences. In developing countries, health and nutritional risks, along with inadequate psychosocial stimulation, hinder cognitive and language development in children under two [125]. Thus, interventions that focus on physical, psychological, and parent–child interactions are crucial for addressing developmental issues in these children [126]. By emphasizing family-centered approaches, these interventions encourage active participation from both the child and caregiver, promoting confidence and potentially preventing future developmental delays.

## 6. Conclusions

In this present review, we highlight the impact of maternal preeclampsia on the long-term development of the offspring’s nervous system, with a particular focus on neuronal development and the underlying mechanisms, including microglial activation, inflammatory responses, and reduced angiogenesis. Moreover, both pharmacological (pravastatin, tadalafil, and melatonin) and non-pharmacological (dietary adjustments, exercise, and standard care) interventions offer promising strategies to mitigate these effects.

Since disruptions in brain development begin during gestation and can persist postnatally, to maximize efficacy, interventions should be initiated prenatally, addressing both the preventive and therapeutic aspects. Ensuring drug safety during pregnancy remains a paramount concern. The complex interplay between maternal preeclampsia and offspring neuronal development underscores the importance of early screening, timely intervention, and rigorous monitoring of drug safety during pregnancy to prevent potential long-term neurological consequences.

## Data Availability

Not applicable.
